# The binding sites of carbon dioxide, nitrous oxide, and xenon reveal a putative exhaust channel for bovine cytochrome *c* oxidase

**DOI:** 10.1016/j.jbc.2025.110395

**Published:** 2025-06-19

**Authors:** Kazumasa Muramoto, Tomohiro Ide, Kyoko Shinzawa-Itoh

**Affiliations:** 1 Department of Life Science, Graduate School of Science, Akoh, Hyogo, Japan; 2 School of Science, University of Hyogo, Akoh, Hyogo, Japan

**Keywords:** cytochrome *c* oxidase, protein structure, X-ray crystallography, oxygen transport, carbon dioxide, nitrous oxide, Xe

## Abstract

Cytochrome *c* oxidase (CcO) catalyzes oxygen (O_2_) reduction at the heme *a*_3_–Cu_B_ site in the transmembrane region of the enzyme. It has been proposed that the hydrophobic channel that connects the transmembrane surface of subunit III through subunit I to the heme *a*_3_–Cu_B_ site is the O_2_ transfer pathway. Gas molecules other than O_2_, including carbon dioxide (CO_2_) generated in the tricarboxylic acid cycle, should also enter the hydrophobic channel, but it is not clear how these molecules are expelled from CcO. We analyzed the crystal structures of CO_2_-, nitrous oxide-, and Xe-bound bovine CcO in the oxidized and reduced states at resolutions of 1.75 to 1.85 Å. Binding of Xe in the channel of subunit I near the interface with subunit III supported the proposed O_2_ transfer pathway. CO_2_, nitrous oxide, and another Xe were all bound to a common site near the branching point of another hydrophobic channel that branched from the O_2_ transport channel. Additional Xe atoms were bound in the second channel leading up to the molecular surface on the intermembrane space side, suggesting that under physiological conditions, CO_2_ that has entered the O_2_ pathway could be passively expelled through this channel. This channel consists of subunit I and nuclear DNA-coded subunit VIIc, suggesting that the addition of subunit VIIc in the process of molecular evolution of mitochondrial CcO has made the CO_2_ exhaust pathway.

Cytochrome *c* oxidase (CcO), a terminal enzyme of the respiratory chain, catalyzes oxygen (O_2_) reduction coupled with the proton pump, generating a proton-motive force across the membrane. CcO is a member of the heme–copper oxygen reductase (HCOR) superfamily found in mitochondria and bacteria. Mammalian CcO consists of three mitochondrial DNA–coded core subunits (subunits I, II, and III) and at least 10 nuclear DNA–coded peripheral subunits (Fig. 1*A*). The electrons from cytochrome *c* are transferred through Cu_A_ in subunit II and heme *a* in subunit I to the O_2_ reduction site, which consists of heme *a*_3_ and Cu_B_. The O_2_ molecule is bound to the reduced heme *a*_3_–Cu_B_ site and then reduced to water molecules using the protons taken up from the mitochondrial matrix. The protons are transferred through the hydrogen-bond networks in the transmembrane α helices of subunit I, referred to as the K and D pathways. The hydrogen-bond network and cavities in the transmembrane α helices surrounding heme *a*, referred to as the H pathway, has been proposed as a proton transfer pathway, although its function remains controversial (1, 2, 3, 4).

**Figure 1:**
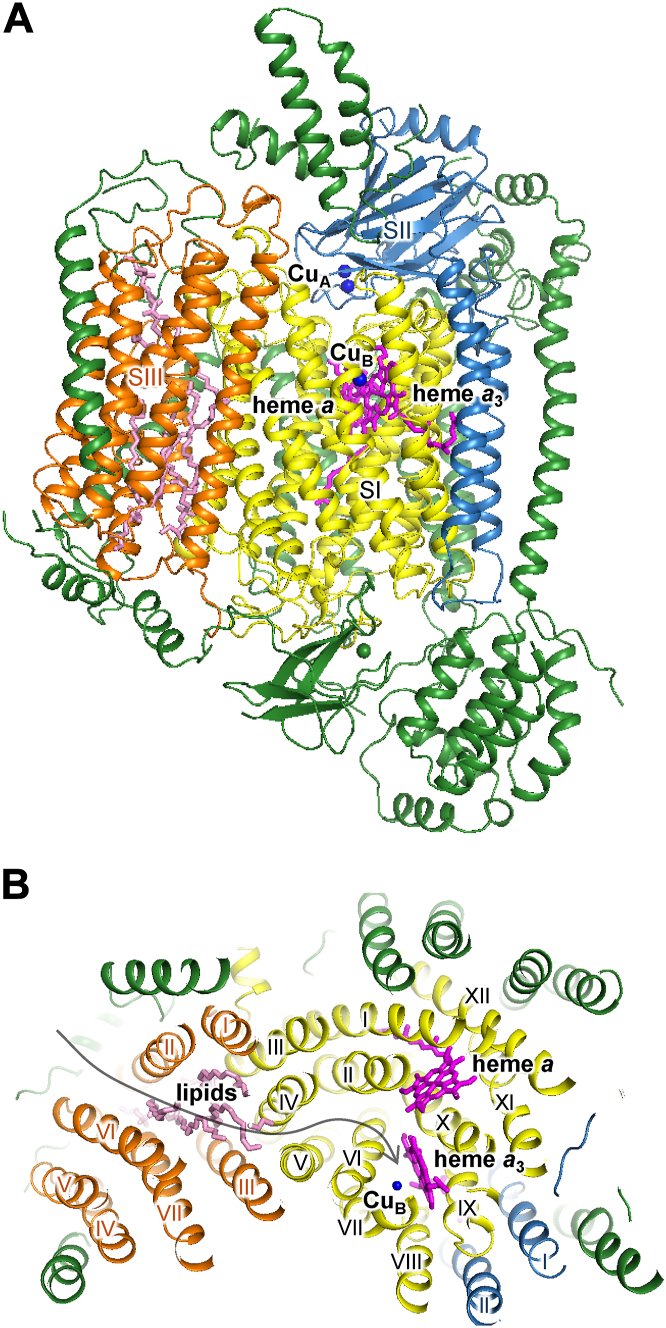
**Whole structure of bovineCcO.***A*, subunits I (residues 1–513), II (residues 1–227), and III (residues 4–261) are colored in *yellow*, *sky blue*, and *orange*, respectively. Subunits IV, Va, Vb, VIa, VIb, VIc, VIIa, VIIb, VIIc, and VIII are colored in *green*. The heme and lipid molecules and the copper atoms are represented by *magenta* and *pink sticks* and by a *blue sphere*, respectively. *B*, cross section of CcO at the level of heme *a*_3_ iron and Cu_B_. The transmembrane helices are labeled with Roman numbers. The *black arrow* indicates the O_2_ transfer pathway. CcO, cytochrome *c* oxidase.

The O_2_ transport channel of bovine CcO has been proposed based on the molecular structure, amino acid modification, and molecular dynamics simulation (5, 6). Between transmembrane helices A and B of subunit I, there is a hydrophobic channel structure that connects to the O_2_ reduction site (Fig. 1*B*). This channel leads through the transmembrane region of subunit III to the surface of CcO in the membrane. A hydrophobic channel structure in subunit III is formed by hydrophobic amino acid residues and lipid side chains, which are bound to the V-valley structure between bundles of two and five transmembrane helices. O_2_ molecules are thought to diffuse through this channel from the transmembrane surface of subunit III to the active center. Previous studies investigated the structure and function of CcO in which the carboxyl group of Glu90 in subunit III next to the hydrophobic channel was modified (7, 8). Modification with dicyclohexylcarbodiimide altered the structure of the lipid side chain bound to the V-shaped valley of subunit III, blocked the hydrophobic channel, and inhibited the enzymatic activity, suggesting that the hydrophobic channel of subunit III functions as an O_2_ transfer pathway (8).

The CcO inhibitors, carbon monoxide, nitric oxide (NO), and cyanide (CN^−^) and azide (N_3_^−^) ions bind to the O_2_ reduction site, indicating that diatomic and triatomic molecules other than O_2_ can also move through the O_2_ channel (9, 10, 11). CN^−^ and N_3_^−^ are probably protonated, and the neutral molecules move through the O_2_ channel. These O_2_ analog molecules have been used as probes to analyze the structural and functional properties of CcO. In addition to these molecules, CO_2_ and nitrous oxide (N_2_O), which may not be ligands at the O_2_ reduction site, affect enzyme function, suggesting that these triatomic molecules can also move through the O_2_ channel. A previous study showed that incubation of the fully oxidized “fast” CcO under a CO_2_ atmosphere caused a 3 nm shift in the Soret spectral peak and slowed the CN^−^ binding rate (12). Therefore, CO_2_, H_2_CO_3_, and/or HCO_3_^−^ are probably bound close to the O_2_ reduction site. Under an N_2_O atmosphere, the activity of isolated CcO was partially and reversibly reduced (13). Infrared spectroscopy showed that N_2_O molecules occupied sites in CcO; however, N_2_O had no observable effect on the Soret peak or on the O_2_ reduction site. Interestingly, infrared spectroscopy detected N_2_O-induced secondary structure changes only in fully reduced CcO and not in fully oxidized CcO. Xe gas has been widely used as a probe for O_2_ to identify the O_2_ transfer pathway. In the HCOR superfamily, the structure of the O_2_ channels of A-type CcO from *Rhodobacter sphaeroides* (*Rs*CcO) and B-type CcO from *Thermus thermophilus* (*Tt*CcO) and the structure of the NO channel of cytochrome *c*-dependent NO reductase from *Pseudomonas aeruginosa* have been proposed based on the Xe-binding sites in their crystal structures (14, 15, 16).

In the O_2_ reduction cycle, molecules other than O_2_ that are present in the mitochondria should also enter the O_2_ channel. However, it is not clear how these molecules are expelled from CcO. In this study, to investigate the properties of the O_2_ channel of mitochondrial CcO, we analyzed the crystal structures of CO_2_-, N_2_O-, and Xe-treated bovine CcO in the fully oxidized and the fully reduced states. We found a common binding site for these molecules in another channel that branched from the O_2_ channel and two Xe-binding sites in the H pathway.

## Results

### Xe-bound structures of bovine CcO

**Figure 2:**
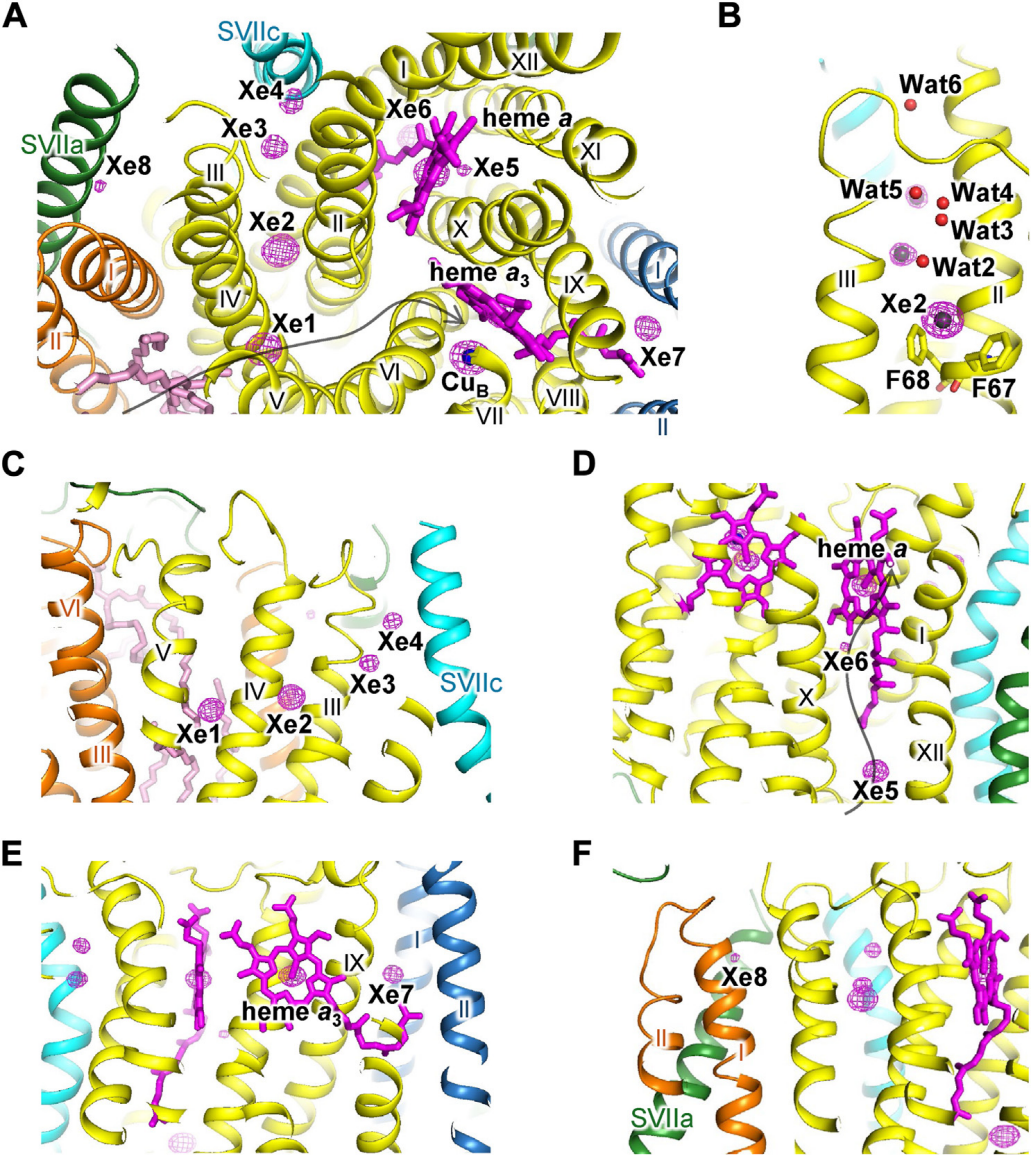
**Structure of the Xe-binding sites in fully reduced CcO under anaerobic conditions**. The anomalous difference map is represented by the *red mesh* contoured at the 8σ level. Subunits I, II, III, and VII, and other subunits are colored in *yellow*, *sky blue*, *orange*, *cyan*, and *green*, respectively. The heme and lipid molecules and Cu_B_ are represented by *magenta* and *pink sticks* and by a *blue sphere*, respectively. *A*, cross section of CcO at the level of heme *a*_3_ iron and Cu_B_. The *black arrow* indicates the O_2_ transfer pathway. *B*–*F*, view from the membrane region. *Top* is the intermembrane side, and *bottom* is the matrix side. *B*, structure around the Xe2 site. The Xe atoms and water molecules (Wat2–Wat6) are represented by *gray* and *red sphere* models, respectively. *C*, structure around the Xe1–Xe4 sites. *D*, structure around the Xe5 and Xe6 sites. The *black arrow* indicates the H pathway. *E*, structure around the Xe7 site. Heme *a*_3_ is represented by a *magenta stick model*. *F*, structure around the Xe8 site. Schematics, detailed structures, and interatomic distances of the Xe-binding sites are shown in Figures S1 and S2 and Table S1, respectively. CcO, cytochrome *c* oxidase.

Since Xe, which is used as a probe for O_2_, exhibits an anomalous scattering effect, it can be observed as peaks in the anomalous difference map by X-ray structural analysis. To identify Xe-binding sites in CcO, we treated dimeric CcO crystals with Xe under the following three conditions. (1) Fully reduced CcO was treated with Xe under an anaerobic environment. (2) Fully oxidized CcO was treated with Xe under an anaerobic environment. (3) Fully oxidized CcO was treated with Xe under an aerobic environment. Using these crystals, we measured the X-ray diffraction data at 1.8 Å resolution. In the analysis of the anomalous difference maps, clear anomalous peaks were observed at eight sites in CcO in the fully reduced states (Fig. 2*A*).

The Xe1 peak was in the O_2_ channel of subunit I near the interface with subunit III. The strongest Xe2 peak was between the two benzene rings of Phe67^I^ and Phe68^I^, which protruded into the space that branched from the O_2_ channel in subunit I (Fig. 2*B*). Xe3–Xe4 were in a hydrophobic channel extended to the molecular surface on the intermembrane space side (Fig. 2*C*). The anomalous peaks of Xe1–Xe4 were significantly lower under aerobic conditions (Table 1), indicating that the binding of Xe1–Xe4 competed with O_2_.

**Table 1:** Peak height in the anomalous difference maps of the Xe-treated CcO crystals

Redox state	Reduced		Oxidized (anaerobic)		Oxidized (aerobic)	
Monomer	A	B	A	B	A	B
Xe1	0.71	0.74	0.62	0.62	0.40	0.42
Xe2	0.80	0.75	0.75	0.72	0.54	0.50
Xe3	0.25	0.21	0.12	0.10	0.04	0.04
Xe4	0.20	0.21	0.16	0.15	0.11	0.11
Xe5	0.63	0.66	0.60	0.56	0.45	0.41
Xe6	0.14	0.13	—	—	—	—
Xe7	0.30	0.31	0.33	0.39	0.21	0.24
Xe8	0.14	0.11	0.17	0.13	0.12	0.07

The values indicate the relative ratio of the anomalous peak height of Xe to that of Cu_B_ in each monomer (A or B) in dimeric CcO. —, binding site for Xe6 is not present.

Xe5 was in the cavity formed by the transmembrane helices (helices X, XI, and XII) of subunit I at about 8 Å from the matrix side surface (Fig. 2*D*). The anomalous peak height of Xe5 was about 80% that of Xe2 and was significantly lower under aerobic conditions (Table 1). In the reduced CcO, Xe6 was in the cavity formed by the redox-dependent structural change of heme *a* side chain (hydroxyfarnesylethyl group) and the transmembrane helices (helices X and XI) of subunit I (Fig. 2*D*). The anomalous peak of Xe6 was much lower than that of Xe5 (Table 1).

Xe7 was in the cavity formed by the terminus of the heme *a*_3_ side chain and the transmembrane helices (helix IX of subunit I and helices I and II of subunit II) at about 8 Å from the transmembrane surface (Fig. 2*E*). Xe8 was near the molecular surface on the intermembrane space side formed by the transmembrane helices (helix III of subunit I, helices I of subunit III, and a single helix of subunit VIIa) (Fig. 2*F*). Xe8 and the tail structure of bound detergent were observed at this site, indicating the replacement of the detergent by Xe8. In Xe7 or Xe8, no significant change in the anomalous peak height was observed under these three conditions (Table 1).

### CO_2_- and N_2_O-bound structures of bovine CcO

**Figure 3:**
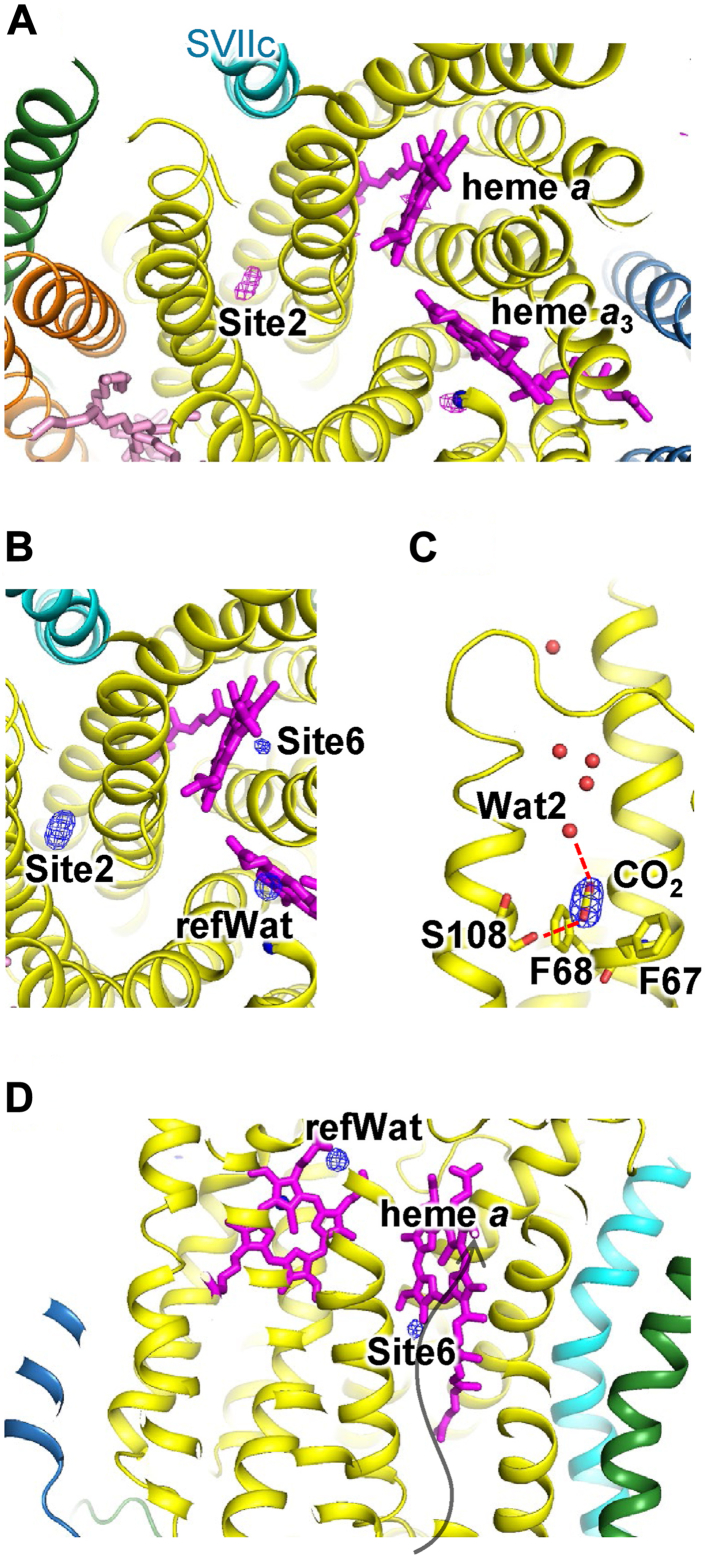
**Structure of the CO_2_-binding sites in fully reduced CcO**. Subunits I, II, III, VIIc, and other subunits are colored in *yellow*, *sky blue*, *orange*, *cyan*, and *green*, respectively. The heme and lipid molecules and Cu_B_ are represented by *magenta* and *pink sticks* and by a *blue sphere*, respectively. *A*, *F*_O_ − *F*_O_ maps are represented by the *red mesh* contoured at the 8σ level. The *black arrow* indicates the O_2_ transfer pathway. *B*–*D*, *F*_O_ − *F*_C_ maps are represented by a *blue mesh* contoured at the 8σ level. CO_2_ and water molecules are represented by *stick* and *red sphere models*, respectively. The hydrogen bonds are represented by *broken red lines*. *Black arrow* indicates the H pathway. refWat indicates the reference water molecule. *A* and *B*, cross section of CcO at the level of heme *a*_3_ iron and Cu_B_. *C* and *D*, view from the membrane region. *Top* is the intermembrane side, and *bottom* is the matrix side. Schematics, detailed structures, and interatomic distances of the CO_2_-binding sites are shown in Figures S1 and S2 and Table S1, respectively. CcO, cytochrome *c* oxidase.

**Figure 4:**
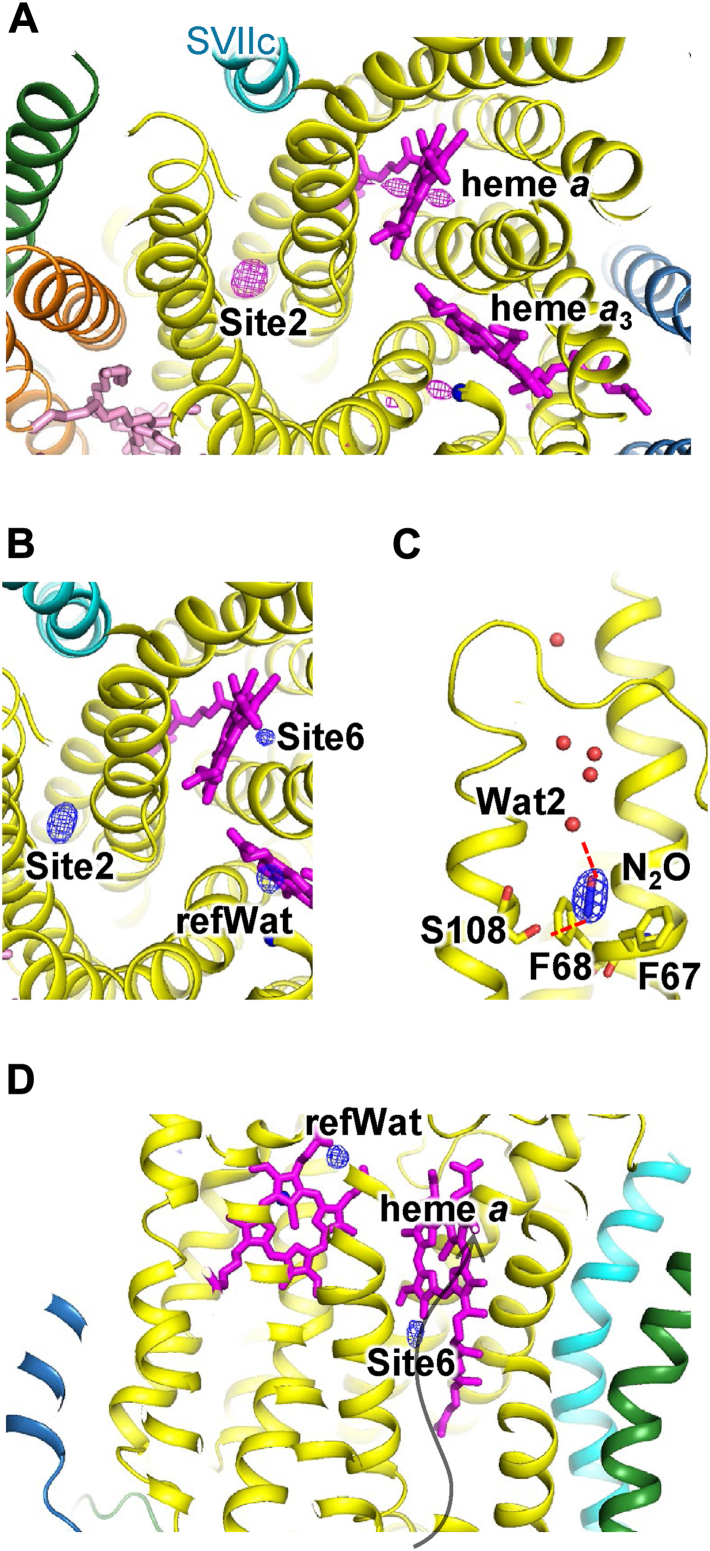
**Structure of the N_2_O-binding sites in fully reduced CcO**. The representation and color of the structural model are the same as in Figure 3. *A*, *F*_O_ − *F*_O_ maps are contoured at the 8σ level. *B*–*D*, *F*_O_ − *F*_C_ maps are contoured at the 8σ level. N_2_O molecule is represented by *stick model*. Schematics, detailed structures, and interatomic distances of the N_2_O-binding sites are shown in Figures S1 and S2 and Table S1, respectively. CcO, cytochrome *c* oxidase; N_2_O, nitrous oxide.

To identify CO_2_- and N_2_O-binding sites, we treated dimeric CcO crystals with CO_2_ or N_2_O in both the fully reduced and the aerobic fully oxidized states. The X-ray diffraction data at 1.75 to 1.85 Å resolution were measured for the CO_2_- and N_2_O-treated CcO crystals and for an untreated CcO crystal in the fully reduced state as a reference. The *F*_O_ − *F*_O_ maps of CO_2_-treated–untreated and N_2_O-treated–untreated CcOs in the fully reduced state showed clear residual electron density in a site (site 2), the same as the Xe2-binding site (Figs. 3*A* and 4*A*). In the *F*_O_ − *F*_C_ maps, elongated residual electron density was observed at site 2 (Figs. 3*B* and 4*B*), and at a site (site 6) the same as the Xe6-binding site (FigS. 3*D* and 4*D*), whereas relatively weak electron density was observed in untreated CcO (Table 2). Therefore, we assigned the electron density to a single molecule of CO_2_ or N_2_O. At site 2, CO_2_ or N_2_O was oriented to form weak hydrogen bonds with both the hydroxyl group of Ser108^I^ and a water molecule (Wat2) (Figs. 3*C* and 4*C*). In the untreated CcO, we assigned the electron density to a water molecule (Wat1), although other molecules, such as nitrogen (N_2_), could not be ruled out.

**Table 2:** Peak heights of CO_2_ and N_2_O in the *F*_O_ − *F*_C_ maps

Redox state	Reduced		Oxidized		Reduced		Oxidized		Reduced	
Ligand	CO_2_		CO_2_		N_2_O		N_2_O		Wat1	
Monomer	A	B	A	B	A	B	A	B	A	B
Site 2	1.01	1.03	1.00	0.80	1.12	1.13	1.04	0.94	0.24	0.27
Site 6	0.60	0.54	—	—	0.64	0.65	—	—	0.36	0.43

The values indicate the relative ratio of the peak height of CO_2_ or N_2_O to that of the reference water molecule bound to the heme *a*_3_ propionate group in each monomer (A or B) in dimeric CcO. The occupancy of the reference water molecule is assumed to be almost full in any condition. —, site 6 is not present.

In the *F*_O_ − *F*_C_ maps of CO_2_- and N_2_O-treated CcO in the fully reduced state, elongated electron density was observed at a site (site 6) the same as the Xe6-binding site (Figs. 3*D* and 4*D*). Because the electron density was significantly stronger than that in untreated CcO (Table 2), it was assigned to CO_2_ or N_2_O. CO_2_ or N_2_O was oriented along and within the elongated spherical cavity of site 6.

## Discussion

### O_2_ transfer pathway

Xe2 was bound in the O_2_ channel of subunit I near the interface with subunit III (Fig. 2*A*). Xe2 probably reached this site from the transmembrane surface of subunit III through the O_2_ channel surrounded by the side chains of lipids bound to the V-shaped valley of subunit III, supporting the idea that O_2_ and other gas molecules are also transferred in the same way. In Xe-untreated fully reduced CcO or previously reported fully oxidized CcO (17), no significant electron density was observed at this site, indicating that unlike Xe, O_2_ and N_2_ were not bound stably to this site. In addition, there was no electron density on other sites of the O_2_ channel, indicating that there was no site where O_2_ bound stably in the channel. This property may be important for the rapid O_2_ transfer to the O_2_ reduction site through this channel (5, 18).

**Figure 5:**
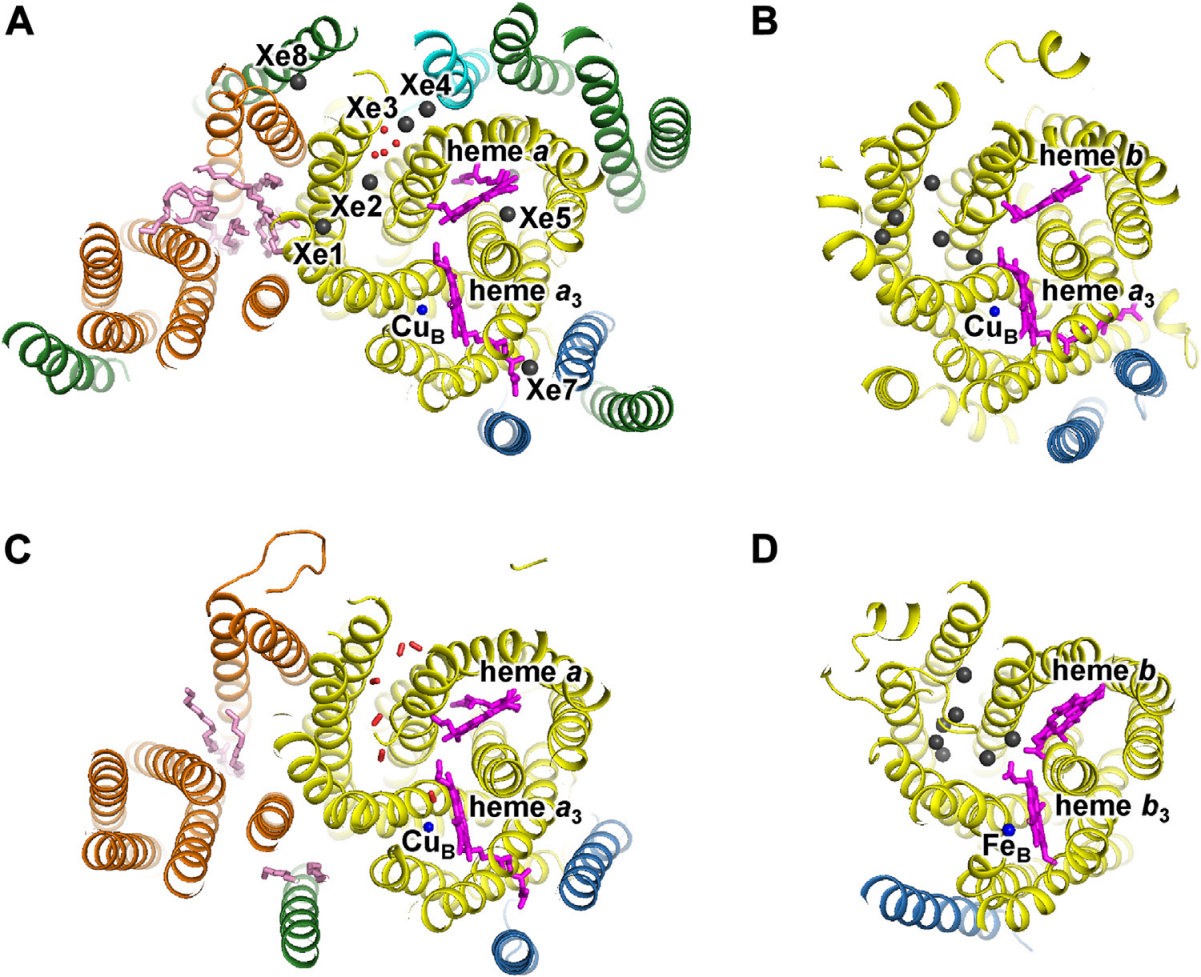
**Structure of the Xe- and O_2_-binding sites in HCOR members**. Structure of the Xe-binding sites of (*A*) bovine CcO (Protein Data Bank [PDB] ID: 9KUM), (*B*) *Tt*CcO (PDB ID: 3S33), and (*D*) *Pa*cNOR (PDB ID: 5GUX). *C*, structure of the O_2_-binding sites in P*d*CcO (PDB ID: 7AU6). Subunits corresponding to subunits I, II, III, and VIIc of bovine CcO are colored in *yellow*, *sky blue*, *orange*, and *cyan*, respectively. Other subunits are *green*. The heme and lipid molecules and the metal atom corresponding to Cu_B_ of bovine CcO are represented by *magenta* and *pink sticks* and by a *blue sphere*, respectively. The Xe atoms and water molecules are represented by *gray and red spheres*, respectively. CcO, cytochrome *c* oxidase; HCOR, heme–copper oxygen reductase; *Pa*cNOR, *Pseudomonas aeruginosa* NO reductase; *Pd*CcO, *Paracoccus denitrificans* CcO; *Tt*CcO, *Thermus thermophilus* CcO.

A structure homologous to the O_2_ channels is also found in HCOR members, although in the members that do not have subunit corresponding to subunit III of bovine CcO, the channel is directly open to the membrane at the transmembrane surface of subunit I (Fig. 5). In *Tt*CcO, Xe was bound to the site corresponding to the Xe2-binding site of bovine CcO (Fig. 5, A and B), and the authors suggested that O_2_ also passed through this site (19). This is consistent with our proposed O_2_ pathway in bovine CcO.

### Putative CO_2_ exhaust pathway

CO_2_, N_2_O, and Xe_2_ were all bound to a common site (site 2) near the branching point of another hydrophobic channel that branched from the O_2_ channel (Figs. 2*A* and 3, *A* and *B*). At site 2, weak electron density was observed in fully reduced CcO (Table 2) and in previously reported fully oxidized CcO (17). Although this peak was assigned to a water molecule, it could be replaced by a gas molecule, such as O_2_ or N_2_. The electron density of CO_2_ or N_2_O at site 2 was remarkably strong and little changed in the aerobic and anaerobic conditions. These results indicated that the affinity for CO_2_ and N_2_O at site 2 was higher than that of O_2_, N_2_, and water molecules. The hydrophobic interactions that sandwiched the ligand between Phe67^I^ and Phe68^I^ may affect the stability of ligands at site 2. These two residues are also conserved in *Rs*CcO, CcO from *Paracoccus denitrificans*, cytochrome *caa*_3_ from *T*. *thermophilus* (20), and cytochrome *bo*_3_ ubiquinol oxidase from *Escherichia coli* (21); thus, these residues might be important for O_2_ transfer in A-type HCOR members. In addition to the hydrophobic interactions, hydrogen bonds between CO_2_ or N_2_O and the hydroxyl group of Ser108^I^, and between CO_2_ or N_2_O and Wat2 may stabilize the binding of CO_2_ and N_2_O.

**Figure 6:**
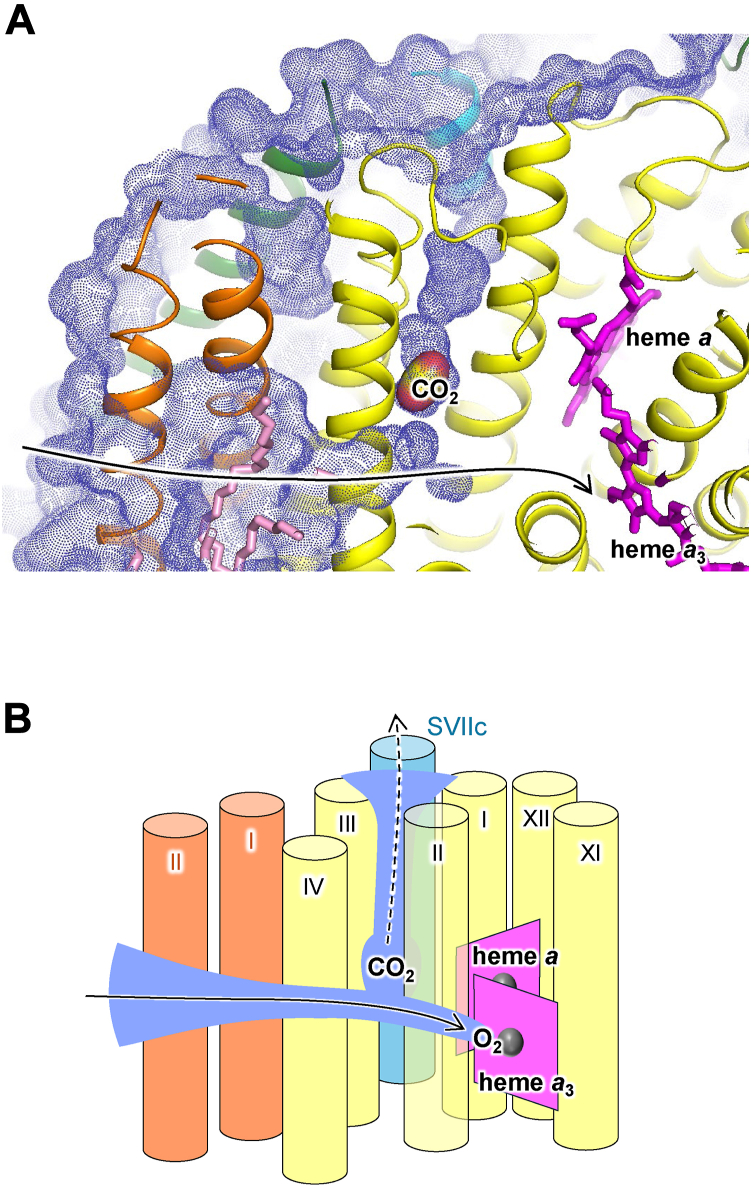
**Molecular surface of CcO and the putative CO_2_ exhaust pathway**. *A*, the molecular surface represented by *blue dots* was calculated based on the structure of the CO_2_-bound reduced CcO with a probe having a radius of 1.3 Å. CO_2_, Wat2–Wat6, and lipids were excluded from the calculation. The *solid arrow* indicates the O_2_ transfer pathway. Subunits I, III, VIIc, and other subunits are colored in *yellow*, *orange*, *cyan*, and *green*, respectively. The heme and lipid molecules are represented by *magenta* and *pink sticks*, respectively. The CO_2_ molecule is represented by van der Waals spheres. *B*, hypothesis of the CO_2_ exhaust channel. The transmembrane helices of subunits I, III, and VIIc are colored in *yellow*, *orange*, and *cyan*, respectively, and labeled with Roman numbers. The channel structure in CcO is represented by *blue region*. The *broken arrow* indicates the putative CO_2_ exhaust pathway. CcO, cytochrome *c* oxidase.

Xe3 and Xe4 were bound in the hydrophobic channel leading from site 2 to the molecular surface on the intermembrane space side (Fig. 2*B*). In Xe-untreated fully reduced or fully oxidized CcO, weak elongated electron density including the Xe3- and Xe4-binding sites was observed (17, 22). This electron density could be assigned to the detergent tail or mobile gas molecules that could be replaced with Xe. In this channel, other electron density peaks were also observed that were different from the Xe-binding sites. Although these peaks were assigned to water molecules (Wat2–Wat6 in Table 3 and Fig. 2), the peak heights were low and there was no typical hydrogen bond, except for Wat5. Therefore, Wat2–Wat4 and Wat6 are not always present. Wat5 could be temporarily replaced by gas molecules moving through the channel. This channel consists of subunit I and nuclear DNA–coded subunit VIIc, indicating that this channel is unique to mitochondrial CcO. In the mitochondrial matrix, CO_2_ is generated in the tricarboxylic acid cycle. Therefore, CO_2_ concentrations should be higher inside the mitochondrial inner membrane than on the outside. When CO_2_ is moved from the mitochondrial matrix to the outside of the cell, CO_2_ that has entered the O_2_ pathway of CcO from the membrane may be trapped at site 2. Based on the above, we propose that CO_2_ bound to site 2 could be passively expelled to the outside of mitochondrial inner membrane through this channel (Fig. 6). However, since the structural analysis of this study shows ligand binding rather than transport, further experimental validation is needed to demonstrate this hypothesis.

**Table 3:** Peak height of possible water molecules in the *F*_O_ − *F*_C_ map

Monomer	A	B
Site 2	0.24	0.27
Wat2	0.29	0.39
Wat3	0.14	0.17
Wat4	0.35	0.45
Wat5	0.80	0.78
Wat6	0.34	0.36

The *F*_O_ − *F*_C_ map of CcO in the fully reduced state was examined. The values indicate the relative ratio of the peak height of possible water molecules to that of the reference water molecule bound to the heme *a*_3_ propionate group in each monomer (A or B) in dimeric CcO. Site 2 is the same as shown in Table 2.

Previous studies reported that the binding of CO_2_ to CcO reduces the reactivity of CcO with CN^−^, and the binding of N_2_O partially inhibits the activity of CcO (12, 13). In this study, we measured the effect of N_2_O on O_2_ consumption activity of CcO. The O_2_ consumption rate slightly decreased in the presence of N_2_O (Fig. S3). Although the binding of CO_2_ or N_2_O to site 2 does not block the O_2_ channel, it may inhibit the diffusion of gas molecules through the putative CO_2_ channel. The channels corresponding to the putative CO_2_ channel in bovine CcO have different structures in bacterial HCOR members (Fig. 5). Because the structures in bacterial HCOR members consist only of core subunits, which lack a subunit corresponding to subunit VIIc of bovine CcO, the channels are open to the membrane region. The binding of Xe corresponding to Xe2 has been reported in *Rs*CcO and *Tt*CcO (Fig. 5*B*) (14, 15). In addition, multiple Xe atoms or the electron density peaks assigned to O_2_ have been observed in this channel of NO reductase from *P*. *aeruginosa* or CcO from *Paracoccus denitrificans*, respectively (Fig. 5, *D* and *C*) (16, 23). Therefore, it has been proposed that the channels in bacterial HCOR members are used for the O_2_ or NO entry pathway (14, 16, 23). However, it is unlikely that the putative CO_2_ channel of bovine CcO is used mainly as the O_2_ entry pathway for the following reasons. First, this channel is open to the intermembrane side, which is less favorable for O_2_ access than being open to the membrane. Second, there are several sites in this channel where gas molecules can be bound, which would hinder the rapid diffusion of O_2_. Third, when CO_2_ is bound to site 2, this channel is blocked, which may inhibit the O_2_ supply. Taken together, these findings suggest that the addition of subunit VIIc in the process of molecular evolution of mitochondrial CcO has changed the O_2_ entry pathway to the CO_2_ exhaust pathway that traps entered CO_2_ and diffuses it to the outside.

### Gas molecule binding to the H pathway

Xe5 was in the cavity near the matrix surface, suggesting that Xe5 moved from the solvent region on the matrix side. This cavity is part of the entry to the H pathway (Fig. 2*D*), which has been proposed to be a proton (hydronium ion, H_3_O^+^) transfer pathway (24). The electron density of Xe5 was strong, indicating that Xe5 was stably bound to the cavity. Because the cavity was mostly occupied by Xe5, Xe5 could inhibit the proton (H_3_O^+^) entry to the H pathway. In native (Xe-untreated) CcO, there was electron density assigned to H_2_O in this cavity. However, another molecule, such as O_2_ or N_2_, may also be bound in this cavity as well as Xe5.

The structure near heme *a* in the H pathway is coupled to the redox state of CcO (25). Therefore, the cavity appeared between the hydroxyfarnesylethyl group of heme *a* and the helix X of subunit I only in fully reduced CcO. Xe6 or possibly CO_2_ or N_2_O was found at site 6 in this cavity (Figs. 2*D* and 3, *D*, and 4D). Previously, infrared spectroscopy detected N_2_O-induced secondary structure changes only in fully reduced CcO and not in fully oxidized CcO (26). This may be consistent with the binding of N_2_O at site 6 in fully reduced CcO. Site 6 was in the transmembrane region and was about 15 Å from the Xe5-binding site and 24 Å from the intermembrane side surface. There was no channel structure for Xe, CO_2_, or N_2_O in the H pathway that connected site 6 to the Xe5-binding site or intermembrane side surface. Therefore, it is unlikely that Xe, CO_2_, or N_2_O entering from the entrance or exit of the H pathway migrated to site 6. Xe, CO_2_, or N_2_O must have entered from the transmembrane surface and moved through a gap in the helix bundle to site 6, which was 9 Å from the transmembrane surface. In a fully reduced native (ligand-untreated) CcO, the weak electron density at site 6 could be assigned to H_2_O, O_2_, or N_2_.

## Experimental procedures

### Preparation of CO_2_-, N_2_O-, and Xe-treated CcO crystals

CcO in the resting oxidized state was purified from bovine heart mitochondria and crystallized as described previously (17). Dimeric CcO was present in the crystal. For the X-ray diffraction experiment under cryogenic conditions, the crystals were soaked in a solution containing 40 mM sodium phosphate buffer (pH 5.7), 0.2% (w/v) decyl-β-d-maltoside (Dojin), 8% (w/v) polyethylene glycol 4000 (Hampton Research), and 40% (v/v) ethylene glycol by gradual replacement of the solution (stepwise soaking in a solution containing ∼1% higher ethylene glycol for 20 min), as described previously (17). The anaerobic solution was made in a capped vial by adding 5 mM glucose, 1 μM glucose oxidase, and 0.5 μM catalase to the aforementioned solution to consume O_2_ and by replacing the air with N_2_ gas. To reduce CcO, the crystals were soaked in the anaerobic solution supplemented with 5 mM sodium dithionite for 40 min. Full reduction of the crystal was confirmed by measuring 604 nm peak of the α-band absorption using a microspectrometer equipped with a multichannel spectrometer (USB2000; Ocean Optics).

To prepare the CO_2_-, N_2_O-, or Xe-containing solutions, the gas was dissolved in the aforementioned solution in a closed container with pressure removal by bubbling of CO_2_ gas generated from dry ice, N_2_O gas (Nissan Chemical), or Xe gas (Japan Air Gases), respectively, for 10 min. To prepare the CO_2_-, N_2_O-, or Xe-treated CcO crystals, the crystals were soaked for 20 min in the gas containing solution at 4 °C under atmospheric pressure. The crystals were frozen with a cryonitrogen stream at 100 K and stored in liquid N_2_.

### X-ray crystallographic analysis

X-ray diffraction intensity data were collected at BL44XU beam line at SPring-8 (Harima). The experimental conditions are summarized in Table S2. Data processing and scaling were performed with the program XDS (version; June 30, 2023) (27). Statistical analyses of the datasets are summarized in Table S2. The structure factor amplitude of the observed data (|*F*_O_|) was calculated by using the CCP4 (version 7.0.076) program CTRUNCATE (version 1.17.29) (28). Atomic coordinates of Protein Data Bank IDs 8H8R and 8H8S were used as an initial model for structural refinement of the fully oxidized and fully reduced CcO forms, respectively (29). The refinement was performed with the program Refmac5 (version 5.8.0253) (30) with anisotropic *B*-factors. The results of the statistical analyses of the structural refinement are summarized in Table S2. The *F*_O_ − *F*_O_, *F*_O_ − *F*_C_, and anomalous difference Fourier maps were calculated by the CCP4 program FFT (version 7.0.076) (31) using |*F*_O_|, the structure factor calculated from the model structure (*F*_C_), and Bijvoet pairs of |*F*_O_| (|*F*(+)| and |*F*(−)|). The anomalous difference maps of Xe-treated CcOs were calculated in the resolution range of 40 to 1.80 Å. The *F*_O_ − *F*_O_ and *F*_O_ − *F*_C_ difference maps were calculated in the resolution range used for the refinement of CO_2_- and N_2_O-treated CcOs. The molecular surface was calculated with the program MS (32). Figures were drawn using the program PyMOL (Schrödinger).

## Data availability

The structure factors and the atomic coordinates of structural models of the Xe-bound oxidized (aerobic), the Xe-bound oxidized (anaerobic), the Xe-bound reduced, the CO_2_-bound oxidized, the CO_2_-bound reduced, the N_2_O-bound oxidized, the N_2_O-bound reduced, and the reduced forms are deposited at the Protein Data Bank (www.rcsb.org) with accession codes of 9KUK, 9KUL, 9KUM, 9IKF, 9IKG, 9IKH, 9IKI, and 9M56, respectively.

## Supporting information

This article contains supporting information (33).

## Conflict of interest

The authors declare that they have no conflicts of interest with the contents of this article.
