# Supplementary material for: The binding sites of carbon dioxide, nitrous oxide, and xenon reveal a putative exhaust channel for bovine cytochrome c oxidase
Source: J Biol Chem. 2025 Jun 19;301(7):110395. doi: 10.1016/j.jbc.2025.110395 (PMC12275195; doi:10.1016/j.jbc.2025.110395)
Supplement: Supplementary Material [file mmc1.docx]

**Supplementary information for**

**Muramoto *et al*. The binding sites of** **carbon dioxide, nitrous oxide, and xenon reveal a putative exhaust channel for bovine cytochrome *c* oxidase**


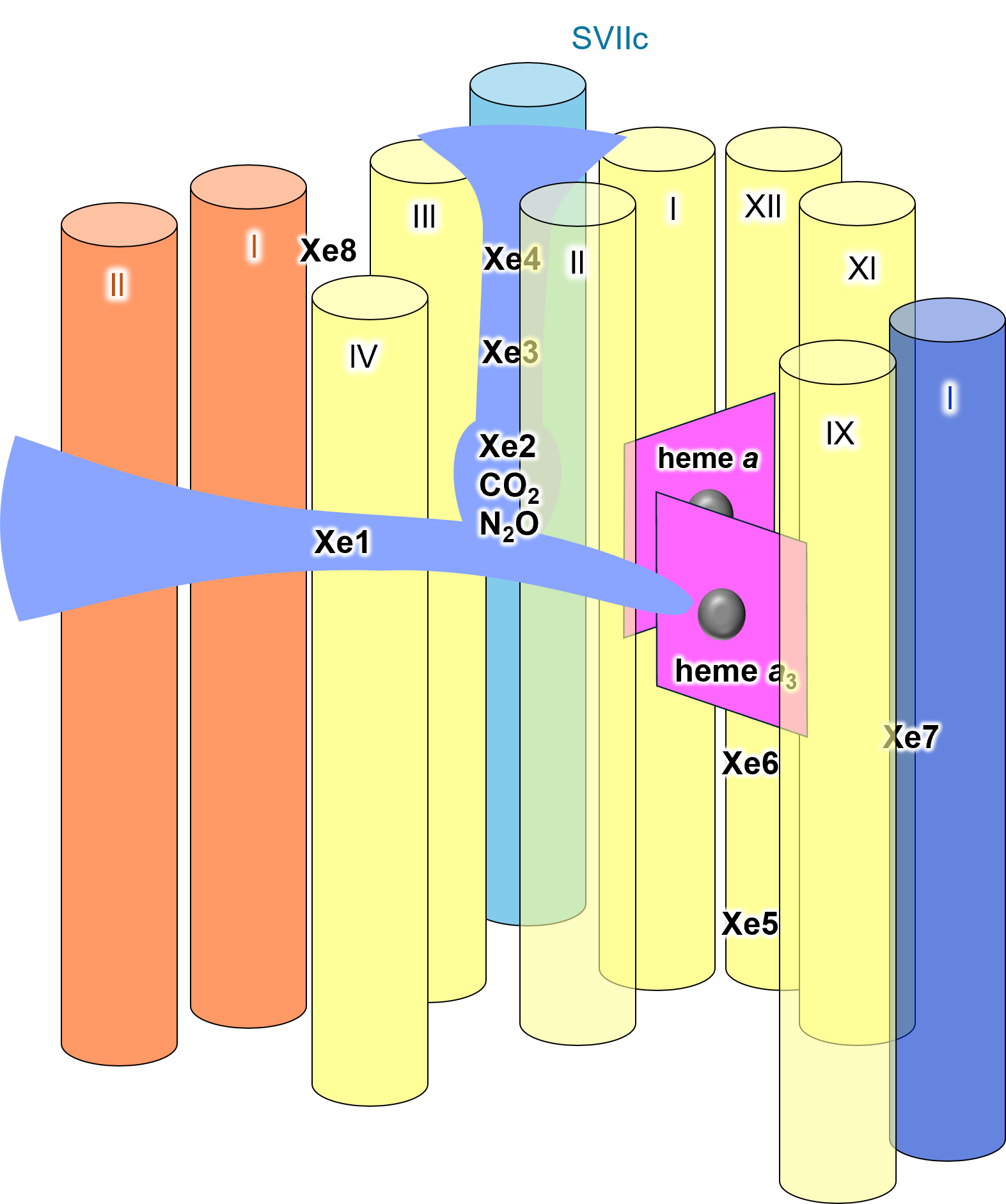


**Figure S1. Schematic representation of the Xe, CO_2_, and N_2_O binding sites.**

The transmembrane helices of subunits I, II, III, and VIIc are colored in yellow, blue, orange, and cyan, respectively. The transmembrane helices are labeled with Roman numbers. The channel structure in CcO is represented by blue region.


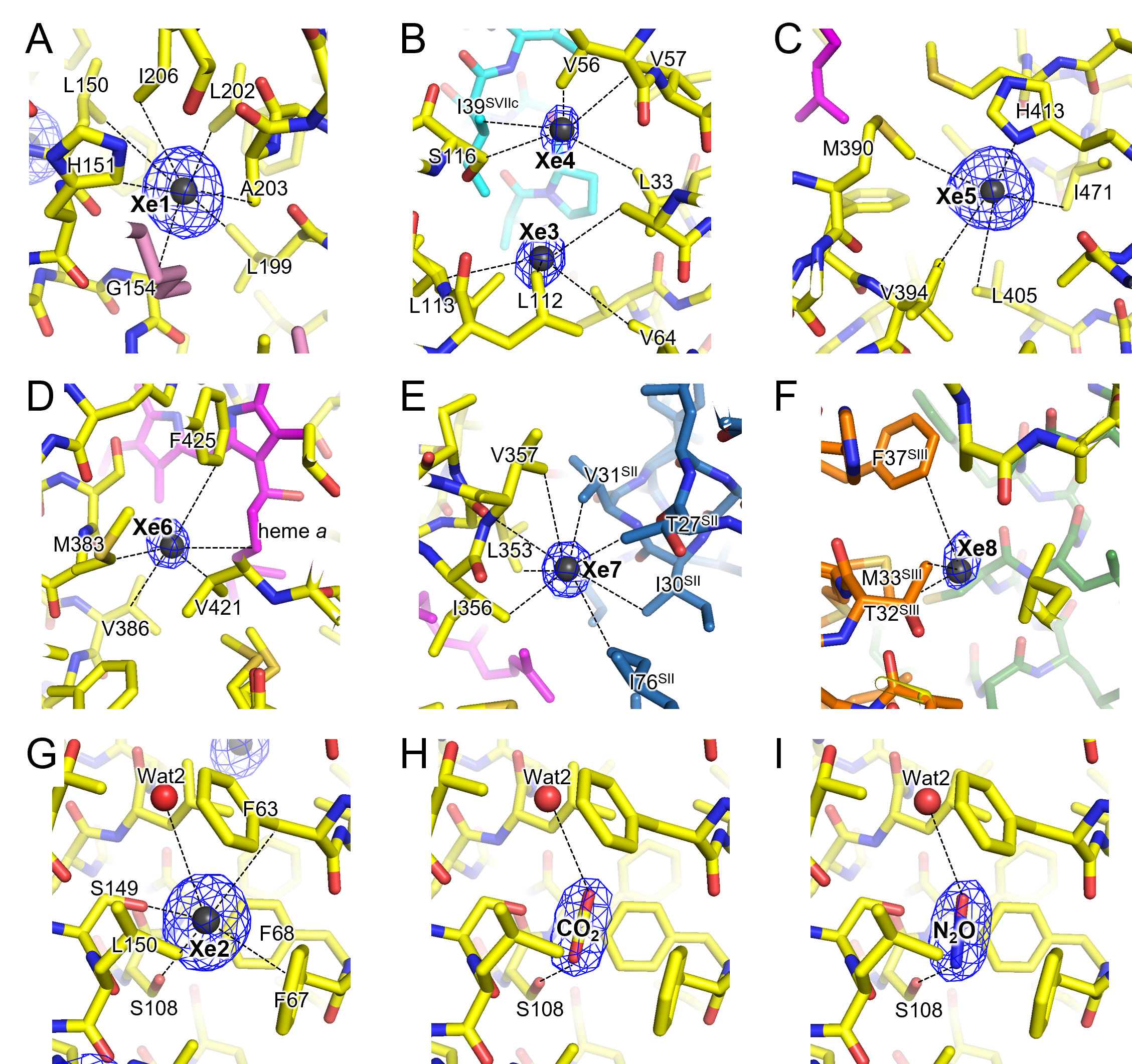


**Figure S2. Detailed structure of the Xe, CO_2_, and N_2_O binding sites.**

Carbon atoms in the subunits I, II, III, VIIc, and other subunits are colored in yellow, sky blue, orange, cyan, and green, respectively. The heme and lipid molecules are represented by magenta and pink sticks, respectively. *F*_O_ − *F*_C_ maps are represented by a blue mesh contoured at the (A-E, G-H) 8σ and (F) 5σ level. (A-G) The Xe atoms and water molecules are represented by gray and red spheres, respectively. (H, I) CO_2_ and N_2_O molecules are represented by stick models. Interatomic distances of broken black lines are shown in Table S1.

**Table S1**

**Interatomic distances of the Xe, CO_2_, and N_2_O binding sites**

| Atom | Atom | Residue | Distance (Å) |  | Atom | Atom | Residue | Distance (Å) |
| --- | --- | --- | --- | --- | --- | --- | --- | --- |
| Xe1 | C^β^ | L150 | 4.8 |  | Xe2 | C^β^ | F63 | 4.2 |
| Xe1 | C^δ2^ | H151 | 4.5 |  | Xe2 | C^β^ | F67 | 3.9 |
| Xe1 | C^α^ | G154 | 4.0 |  | Xe2 | C^ζ^ | F68 | 3.8 |
| Xe1 | C^δ2^ | L199 | 4.4 |  | Xe2 | O^γ^ | S108 | 4.4 |
| Xe1 | C^δ2^ | L202 | 3.8 |  | Xe2 | O^γ^ | S149 | 3.2 |
| Xe1 | C^β^ | A203 | 3.9 |  | Xe2 | C^δ2^ | L150 | 3.6 |
| Xe1 | C^δ1^ | I206 | 3.9 |  | Xe2 | O | Wat2 | 4.8 |
|  |  |  |  |  |  |  |  |  |
| Xe3 | Xe4 |  | 5.2 |  | O1 (CO_2_) | O^γ^ | S108 | 3.5 |
| Xe3 | C^γ2^ | V64 | 4.4 |  | O2 (CO_2_) | O | Wat2 | 3.9 |
| Xe3 | C^δ2^ | L112 | 3.6 |  |  |  |  |  |
| Xe3 | C^α^ | L113 | 4.0 |  | N (N_2_O) | O^γ^ | S108 | 3.5 |
|  |  |  |  |  | O (N_2_O) | O | Wat2 | 3.9 |
| Xe4 | C^δ2^ | L33 | 3.8 |  |  |  |  |  |
| Xe4 | C^γ1^ | V56 | 4.2 |  |  |  |  |  |
| Xe4 | C^γ2^ | V57 | 4.0 |  |  |  |  |  |
| Xe4 | C^β^ | S116 | 4.2 |  |  |  |  |  |
| Xe4 | C^γ2^ | I39^SVIIc^ | 3.5 |  |  |  |  |  |
|  |  |  |  |  |  |  |  |  |
| Xe5 | C^ε^ | M390 | 4.2 |  |  |  |  |  |
| Xe5 | C^γ2^ | V394 | 4.4 |  |  |  |  |  |
| Xe5 | C^γ1^ | L405 | 4.6 |  |  |  |  |  |
| Xe5 | N^γ1^ | H413 | 4.3 |  |  |  |  |  |
| Xe5 | C^γ2^ | I471 | 3.8 |  |  |  |  |  |
|  |  |  |  |  |  |  |  |  |
| Xe6 | C^13^ | heme *a* | 3.7 |  |  |  |  |  |
| Xe6 | C^α^ | M383 | 3.8 |  |  |  |  |  |
| Xe6 | C^β^ | V386 | 4.4 |  |  |  |  |  |
| Xe6 | C^γ1^ | V421 | 3.7 |  |  |  |  |  |
| Xe6 | C^ε2^ | F425 | 4.5 |  |  |  |  |  |
|  |  |  |  |  |  |  |  |  |
| Xe7 | O | L353 | 4.0 |  |  |  |  |  |
| Xe7 | C^δ2^ | L353 | 4.2 |  |  |  |  |  |
| Xe7 | C^γ2^ | I356 | 3.6 |  |  |  |  |  |
| Xe7 | C^γ2^ | V357 | 4.5 |  |  |  |  |  |
| Xe7 | C^γ2^ | T27^SII^ | 4.1 |  |  |  |  |  |
| Xe7 | C^γ2^ | I30^SII^ | 4.3 |  |  |  |  |  |
| Xe7 | C^γ2^ | V31^SII^ | 3.1 |  |  |  |  |  |
| Xe7 | C^δ1^ | I76^SII^ | 4.0 |  |  |  |  |  |
|  |  |  |  |  |  |  |  |  |
| Xe8 | C^γ2^ | T32^SIII^ | 4.1 |  |  |  |  |  |
| Xe8 | C^ε^ | M33^SIII^ | 3.4 |  |  |  |  |  |
| Xe8 | C^ε2^ | F37^SIII^ | 4.2 |  |  |  |  |  |


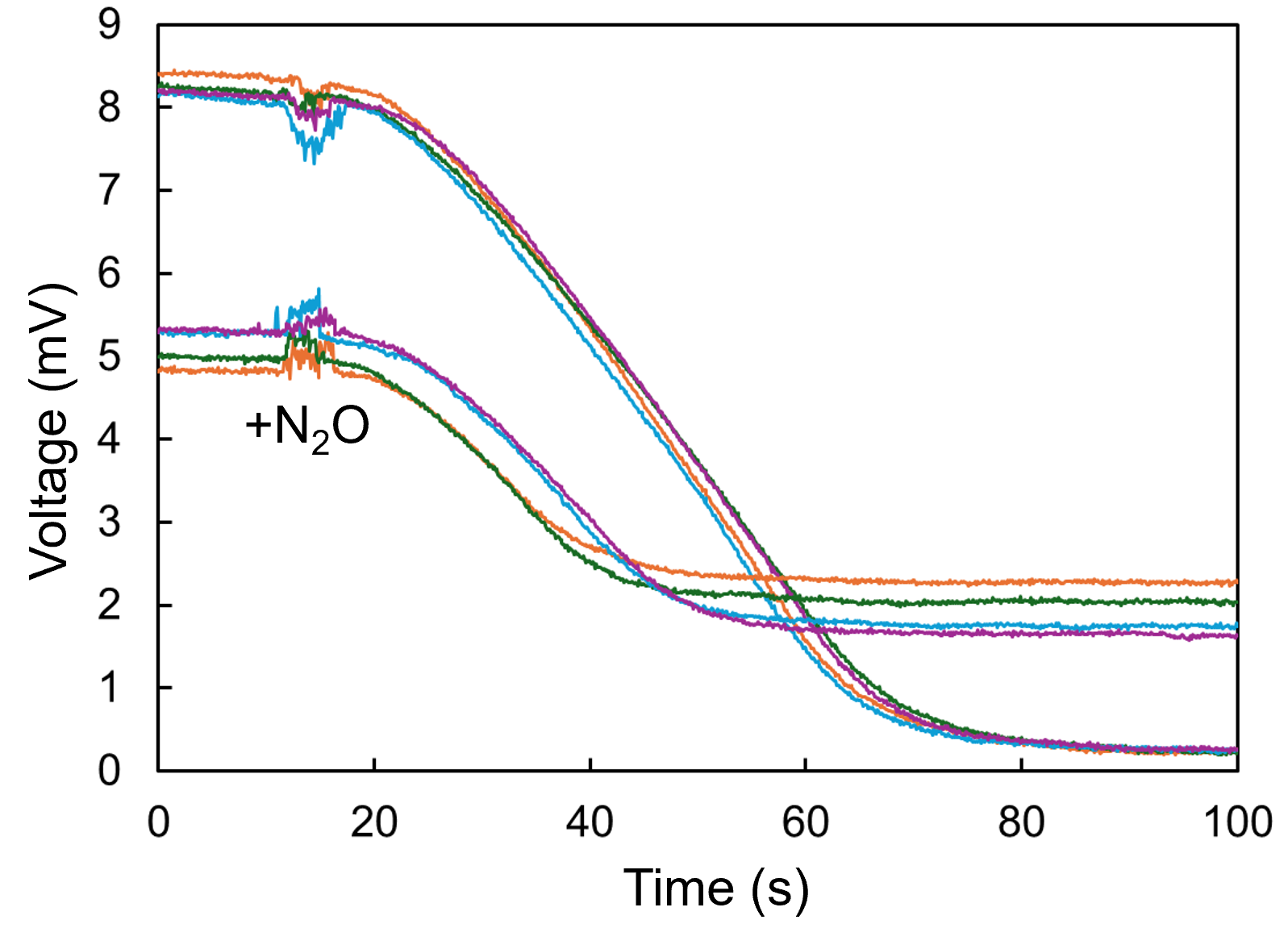


**Figure S3. The O_2_ consumption of CcO in the presence of N_2_O.**

The O_2_ consumption of CcO is measured by the Clark electrode (Rank Brothers Ltd). A circulating water at 20 °C was used to control the temperature of the reaction chamber. The enzymatic reaction was initiated with stirring by addition of 20 μL of 68 μM CcO solution to 2 mL of 100 mM sodium phosphate buffer, pH7.4 supplemented with 0.1% (w/v) decyl-β-D-maltoside (Dojin), 5 mM sodium ascorbate, 2.5 mM *N,N,N',N'*-Tetramethyl-*p*-phenylenediamine dihydrochloride, and 20 μM cytochrome *c* (horse heart). To prepare the reaction solution containing N_2_O, N_2_O gas (Nissan Chemical) was bubbled into the above buffer for 5 minutes. The electrode was connected to a self-built electrical circuit and the computer system with an analog input/output board and software C-LOGGER (CONTEC). 0.7 V of the polarization voltage was applied to the electrode and the change of amplified signal voltage was monitored.

Measurements were performed four times (orange, green, blue, and purple lines) each in the presence and absence of N_2_O. In the absence of N_2_O, voltage of 8.3 mV corresponded to 276 μM O_2_, which was O_2_ concentration in air saturated water at 20 °C. In the presence of N_2_O, based on the assumption that the sensitivity of electrode for N_2_O is 10-fold lower than for O_2_ (1), voltage of 2.0 mV at 100 sec corresponded to 582 μM N_2_O, and voltage of 5.0 mV at 0 sec corresponded to 108 μM O_2_ and 582 μM N_2_O. The data between 30 and 35 sec were used to calculate the O_2_ reduction rate. The average O_2_ reduction rates in the presence and absence of N_2_O were 6.5 O_2_/s (26 e^−^/s) and 7.5 O_2_/s (30 e^−^/s), respectively.

(1): Pouvreau, L. A. M. Strampraad, M. J. F. Van Berloo, S. Kattenberg, J. H. de Vries, S. (2008) NO, N_2_O, and O_2_ reaction kinetics: scope and limitations of the Clark electrode. *Methods. Enzymol.* **436**, 97-112 doi: 10.1016/S0076-6879(08)36006-6.

**Table S2**

**Experimental conditions, diffraction data, and refinement statistics**

| Redox state | Reduced | Oxidized (anaerobic) | Oxidized  (aerobic) | Reduced | Oxidized | Reduced | Oxidized | Reduced |
| --- | --- | --- | --- | --- | --- | --- | --- | --- |
| Ligand | Xe | Xe | Xe | CO_2_ | CO_2_ | N_2_O | N_2_O | Untreated |
| Experimental conditions |  |  |  |  |  |  |  |  |
| Temperature (K) | 100 | 100 | 100 | 100 | 100 | 100 | 100 | 100 |
| Wavelength (Å) | 1.0 | 1.0 | 1.0 | 0.9 | 0.9 | 0.9 | 0.9 | 0.9 |
| Diffraction data |  |  |  |  |  |  |  |  |
| Unit cell (*a*,*b*,*c*) (Å) | 182.5, 204.7, 177.8 | 181.9, 204.0, 178.0 | 181.9, 204.0, 178.0 | 182.7, 205.1, 177.6 | 182.5, 204.4, 177.8 | 181.9, 204.0, 177.7 | 182.1, 204.3, 177.8 | 181.8, 204.1, 177.8 |
| Resolution (Å) | 200–1.80 (1.85–1.80) | 200–1.80 (1.85–1.80) | 200–1.80 (1.85–1.80) | 200–1.85 (1.90–1.85) | 200–1.75 (1.80–1.75) | 200–1.75 (1.80–1.75) | 200–1.75 (1.80–1.75) | 200–1.60 (1.64–1.60) |
| Observed reflections | 25,242,642 (1,765,712) | 25,089,963 (1,754,676) | 16,767,939 (1,168,876) | 19,431,880 (1,336,945) | 27,441,661 (2,029,613) | 45,968,548 (3,455,472) | 18,332,011 (1,386,885) | 35,980,784 (2,713,067) |
| Independent reflections | 1,192,569 (88,326) | 1,185,920 (87,780) | 1,185,899 (87,777) | 1,100,575 (81,449) | 1,295,927 (96,102) | 1,288,078 (95,368) | 1,292,429 (95,800) | 1,686,605 (125,030) |
| Redundancy | 21.2 (20.0) | 21.2 (20.0) | 14.1 (13.3) | 17.7 (16.4) | 21.2 (21.1) | 14.0 (14.0) | 14.2 (14.5) | 21.3 (21.7) |
| Completeness (%) | 100 (100) | 100 (100) | 100 (100) | 100 (100) | 100 (100) | 100 (99.9) | 100 (100) | 100 (100) |
| <*I*/σ> | 28.61 (1.74) | 38.79 (4.48) | 31.52 (3.46) | 15.7 (1.31) | 18.5 (1.32) | 22.2 (1.25) | 14.1 (1.19) | 19.43 (1.17) |
| *R*_meas_ | 0.067 (2.34) | 0.051 (0.86) | 0.053 (0.98) | 0.117 (2.43) | 0.108 (2.70) | 0.118 (3.94) | 0.113 (2.91) | 0.059 (2.28) |
| *CC*_1/2_ (%) | 100 (78.8) | 100 (96.4) | 100 (92.7) | 99.9 (74.1) | 99.9 (76.7) | 100 (74.8) | 99.9 (69.5) | 100 (56.4) |
| Wilson B factor (Å^2^) | 33.3 | 28.9 | 28.1 | 31.8 | 30.5 | 31.1 | 29.3 | 29.2 |
| Refinement |  |  |  |  |  |  |  |  |
| Resolution (Å) | 40–1.80 (1.85–1.80) | 40–1.80 (1.85–1.80) | 40–1.80 (1.85–1.80) | 40–1.85 (1.90–1.85) | 40–1.75 (1.80–1.75) | 40–1.75 (1.80–1.75) | 40–1.75 (1.80–1.75) | 40–1.60 (1.64–1.60) |
| *R* | 0.14 (0.28) | 0.13 (0.13) | 0.12 (0.15) | 0.13 (0.28) | 0.13 (0.28) | 0.15 (0.33) | 0.14 (0.29) | 0.13 (0.30) |
| *R*_free_ | 0.18 (0.30) | 0.16 (0.19) | 0.16 (0.20) | 0.17 (0.29) | 0.17 (0.29) | 0.19 (0.34) | 0.17 (0.30) | 0.15 (0.30) |
| r.m.s.d., bonds (Å) | 0.016 | 0.016 | 0.016 | 0.015 | 0.015 | 0.017 | 0.016 | 0.016 |
| r.m.s.d., angles (degrees) | 1.9 | 1.8 | 1.8 | 1.9 | 1.9 | 2.1 | 2.0 | 1.9 |

The values in parentheses represent data for the highest resolution shell.
